# Vitamin D receptors (VDR), hydroxylases CYP27B1 and CYP24A1 and retinoid-related orphan receptors (ROR) level in human uveal tract and ocular melanoma with different melanization levels

**DOI:** 10.1038/s41598-019-45161-8

**Published:** 2019-06-24

**Authors:** Anna Markiewicz, Anna A. Brożyna, Ewa Podgórska, Martyna Elas, Krystyna Urbańska, Anton M. Jetten, Andrzej T. Slominski, Wojciech Jóźwicki, Jolanta Orłowska-Heitzman, Grzegorz Dyduch, Bożena Romanowska-Dixon

**Affiliations:** 1 Department of Ophthalmology and Ocular Oncology, Medical College, Jagiellonian University in Kraków, 31-501 Kraków, Poland; 2 Department of Human Biology, Faculty of Biology and Environmental Protection, Nicolaus Copernicus University, 87-100 Toruń, Poland; 3 Department of Biophysics, Faculty of Biochemistry, Biophysics and Biotechnology, Jagiellonian University in Kraków, 31-007 Kraków, Poland; 4 Cell Biology Section, Immunity, Inflammation, and Disease Laboratory, National Institute of Environmental Health Sciences, National Institutes of Health, Research Triangle Park, NC, 27709 USA; 5 Department of Dermatology, Comprehensive Cancer Center, Cancer Chemoprevention Program, University of Alabama at Birmingham, Birmingham, AL 35294 USA; 6 VA Medical Center, Birmingham, AL 35294 USA; 7 Department of Tumor Pathology and Pathomorphology, Oncology Centre - Prof. Franciszek Łukaszczyk Memorial Hospital, Bydgoszcz, Poland; 8 Department of Tumor Pathology and Pathomorphology, Faculty of Health Sciences, Nicolaus Copernicus University Collegium Medicum in Bydgoszcz, 85-796 Bydgoszcz, Poland; 9 Clinical and Experimental Pathomorphology, Jagiellonian University, Medical College, 31-531 Kraków, Poland

**Keywords:** Eye cancer, Predictive markers, Prognostic markers, Outcomes research, Eye cancer

## Abstract

In recent years, a significant number of studies have investigated the preventive role of vitamin D in a number of different neoplasms. In this study, we analyze various components of the vitamin D signaling pathways in the human uveal tract and uveal melanoma, including analysis of the expression of vitamin D receptors (VDR), the activating and inactivating hydroxylases, respectively, CYP27B1 and CYP24A1, and the retinoic acid-related orphan receptors (ROR) α (RORα) and γ (RORγ) in these tissues. We further analyzed the expression of VDR, CYP27B1, CYP24A1, and ROR in relation to melanin levels, clinical stage and prognosis. Our study indicated that the uveal melanoma melanin level inversely correlated with VDR expression. We further showed that vitamin D is metabolized in uveal melanoma. This is significant because until now there has been no paper published, that would describe presence of VDR, hydroxylases CYP27B1 and CYP24A1, and RORα and RORγ in the human uveal tract and uveal melanomas. The outcomes of our research can contribute to the development of new diagnostic and therapeutic methods in uveal tract disorders, especially in uveal melanoma. The presented associations between vitamin D signaling elements and uveal melanoma in comparison to uveal tract encourage future clinical research with larger patients’ population.

## Introduction

Melanomas developing from ocular tissues make up to 5% of all melanomas. Among them, 5% are conjunctival melanomas, 85% uveal melanomas, and 10% are lesions occurring in other ocular structures^1^.

Uveal melanoma is the most frequent primary intraocular tumor in adults. The most frequent location in uveal melanomas is the choroid (90%), less frequent – the ciliary body (6%), while the iris is the least frequent location (4%)^2^. The prevalence varies depending on the population studied: in the USA, this is 5.1 new cases per 1 million inhabitants per year^3,4^. In Europe, the prevalence of this tumor is determined by latitude: in the countries of Southern Europe (Italy, Spain) these are 2 cases per 1 million per year, whilst in the Northern part of the continent (Norway, Denmark) – 8^4,5^. In Asia and Africa, the prevalence is assessed to be about 0.2 to 0.3 new cases per 1 million per year^3,6^. This type of cancer is the most frequent among Caucasians (98%)^3,7^. Uveal melanoma may occur at any age, yet it is quite rare among children and young adults^8^. The incidence increases with age, reaching a constant level at the age of 75, whereas the average age when this cancer is diagnosed ranges from 59 to 62^2–4^.

Thanks to the current methods of uveal melanoma treatment, the rate of positive effects of local treatment is about 76–100%^9,10^. Unfortunately, there are still no effective treatment methods which would allow to cure the disease completely or to prolong significantly the survival of the patients in whose case tumor spread occurred. An average survival period from the moment of diagnosing distant metastases is 4–15 months, while only 10–15% patients survive 1-year^11,12^. The 5-year mortality in patients with uveal melanoma is 31%, while the 15-year mortality is 45%^13^. This brings the necessity of a farther search for new mechanisms which affect the oncogenesis of this tumor, its metastases and also for the prognostic markers allowing better assessment of disease outcome and facilitating the selection of the proper treatment and follow-up pattern.

Within the last few years, the number of studies concerning the function of vitamin D and its effect on human disease has significantly increased. Vitamin D is a prohormone that is produced in the skin through photochemical transformation of 7-dehydrocholesterol upon exposure to ultraviolet radiation B (UVB)^14–19^. Vitamin D is activated through sequential hydroxylations at C25 (by CYP27A1 and/or CYP2R) with following hydroxylation at C12 by CYP27B1 and inactivated by CYP24A1 through hydroxylation at C24 with further shortening at the side chain to produce calcitroic acid^14–17^. Via its interaction with the vitamin D receptor (VDR) active form of vitamin D_3_, 1,25-dihidroxyvitaminD_3_ (1,25(OH)_2_D_3_) regulates the transcription of many genes^16,17,20^. In addition to its role in the regulation of calcium and phosphate metabolism, vitamin D has many other functions that include the regulation of inflammation and immune functions (inhibiting autoimmune processes), stimulation of cell differentiation and maturation, inhibition of cell proliferation, regulation of endocrine functions (e.g., insulin secretion). In addition, It has been reported to stimulate neuron regeneration, increase muscle mass and strength, stimulate hepatic cell regeneration, inhibit the development of depression and schizophrenia, affect the circulatory system through renin-angiotensin-aldosterone system, and decrease the risk of oral decay^21–23^.

Another important functions of active form of vitamin D are its anti-tumor effects, antimutagenic properties (e.g. protection against the effect of free radicals) and the regulation of tumor proliferation, apoptosis, and angiogenesis^24,25^.

It was shown that vitamin D deficiency or dysregulated vitamin D signaling can play an important role in oncogenesis, clinical advancement and prognosis in such neoplasms as cutaneous melanomas, bladder, breast, lung, ovarian, pancreatic, thyroid, prostate and colorectal cancers^26–36^. In addition, amounts in expression of its receptor (VDR), expression of hydroxylases participating in its activation and metabolism (CYP27B1, CYP24A1), as well as the expression of the alternative receptors, retinoic acid-related orphan receptors α and γ (RORα and RORγ) in tumor tissues can affect the outcome of the disease^26–29,31–37^.

Because of such versatile actions of vitamin D, more and more scientists have taken up the research concerning its function in ophthalmologic diseases. Decreased levels of vitamin D metabolites in blood serum have been linked to an increased risk of age-related macular degeneration (AMD), dry eye syndrome, diabetic retinopathy and impaired corneal healing after surgeries and traumas^38–42^.

The studies carried out by Alsalem *et al*. confirmed the presence of VDR and CYP27A1, CYP2R1, CYP27B1, CYP24A1 in the cells of the protective barrier of human eye, i.e. primary human scleral fibroblasts (HSF), in cultured corneal endothelial cells (HCEC-12), adult retinal pigment epithelial (ARPE-19), and nonpigmented ciliary body epithelial cells (ODM-2)^43^.

Other receptors linked to the control of cellular functions by sterols^44–46^, including vitamin D^47,48^ and lumisterol compounds^49^, are the nuclear receptors RORα and RORγ. These transcription factors regulate several physiological and pathological processes, including various immune and endocrine functions, as well as cell growth, differentiation, apoptosis, and cancer^50–56^. RORα and RORγ also participate in the regulation of circadian rhythms by directly regulating the expression of a number of clock genes^57,58^.

New vitamin D hydroxyderivatives that can also act as inverse agonists on ROR are produced *ex-vivo* by adrenal and placenta fragments, epidermal keratinocytes and are detectable in human epidermis and serum^59–61^. It has been recently demonstrated that RORα and RORγ are expressed in normal and pathological human skin and that hydroxyderivatives of vitamin D can act as inverse agonists of ROR^47,49,54^. In cutaneous melanoma a inverse correlation between the RORα and RORγ expression and the progression of human cutaneous melanoma has been observed^62^.

To fill the gap in our knowledge on vitamin D activity in uveal melanoma, we decided to evaluate the expression pattern of the VDR, CYP27B1 and CYP24A1 hydroxylases and RORα and RORγ in the cells of uveal melanoma, in normal melanocytes and in other normal uveal cells in humans. Additionally, the relationship between VDR, CYP27B1, CYP24A1, RORα and RORγ expression, with clinical presentation and melanin content were examined.

## Results

### Expression of the VDR in human uveal tract and uveal melanoma

**Figure 1:**
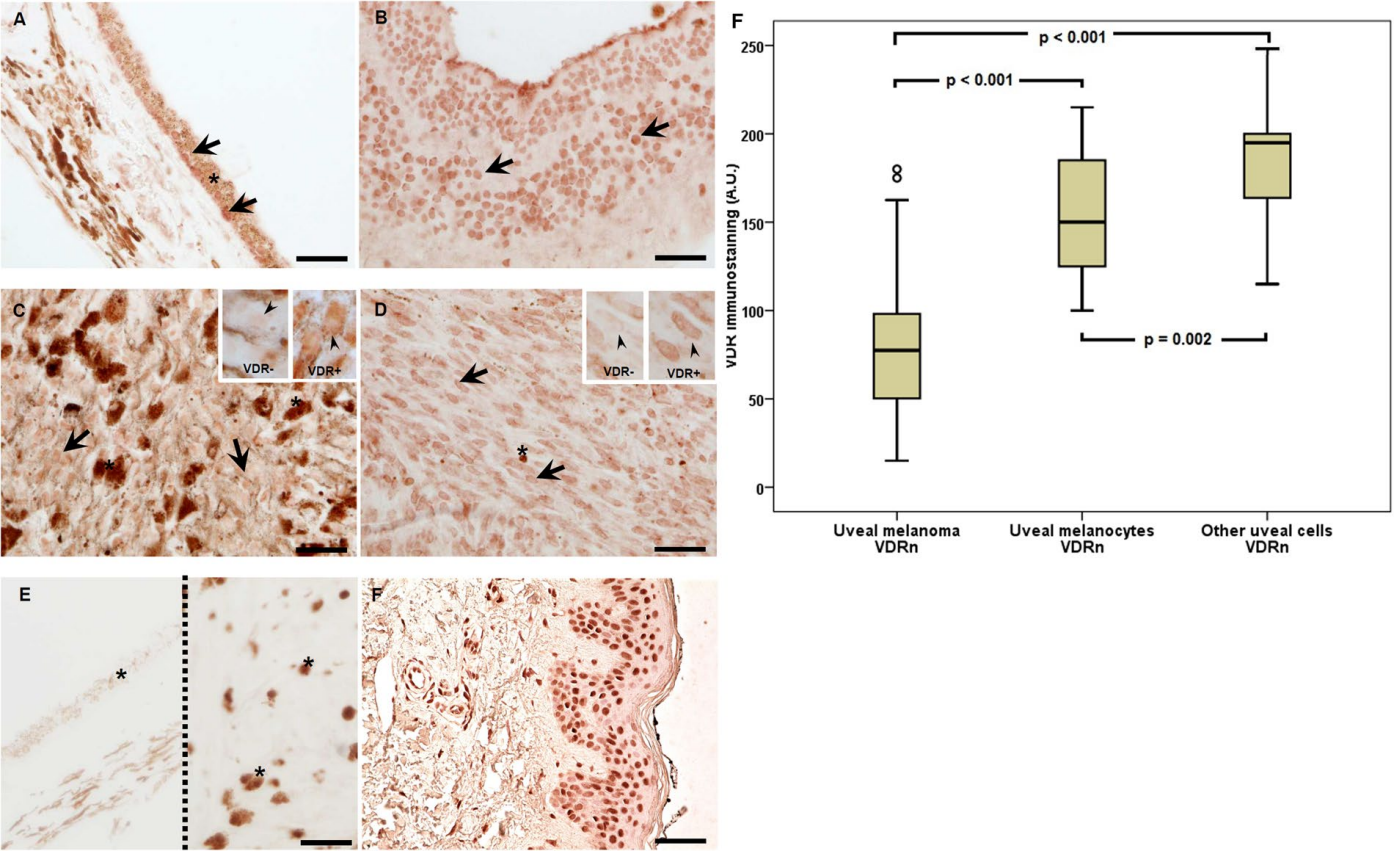
Representative VDR immunostaining in uveal pigmented cells (**A**), other cells (**B**), strongly pigmented (**C**) and slightly pigmented (**D**) melanomas and negative (**E**; two separate samples separated with dotted line) and positive (**F**) controls. Inserts present high magnification of negative (VDR−) and positive (VDR+) cell nuclei (as indicated by arrow heads). Arrows indicate nuclear VDR immunostaining, asterisks indicate melanin, scale bar: 50 μm. Mean nuclear VDR immunolabelling (A.U. – arbitrary units) in melanoma, normal melanocytes and other normal uveal cells (**F**) (statistically significant differences are denoted with the Wilcoxon signed-rank test).

Similarly as in the cells of cutaneous melanoma, VDR immunostaining has been found in uveal melanoma both in cell nuclei (VDRn) and in cytoplasm (VDRc). The presence of nuclear vitamin D receptor (VDRn) was observed in all types of the uveal tissues of adult patients. VDRn was present both in the tumor cells (uveal melanoma, 100%), and in non-tumor cells, such as normal melanocytes (uveal melanocytes, 96.77%) and other normal cells of the surrounding connective tissue (fibroblasts, 70.97%) (Fig. 1A–E). The difference in the number of evaluated receptors between normal and tumor tissue was statistically significant. In the tumor cells, the level of VDRn was significantly lower (the Wilcoxon signed-rank test, p < 0.001). The analysis showed also a statistically significant difference between the numbers of VDRn in normal cells of the uveal tissue. Melanocytes contained less VDRn than other cells of connective tissue did (the Wilcoxon signed-rank test, p = 0.002) (Fig. 1F). In uveal melanoma, the presence of cytoplasmic vitamin D receptors (VDRc) was found (Me = 25.0, and Q1 = 10, Q3 = 43.75).

### CYP24A1 and CYP27B1 in human uveal tract and uveal melanoma

**Figure 2:**
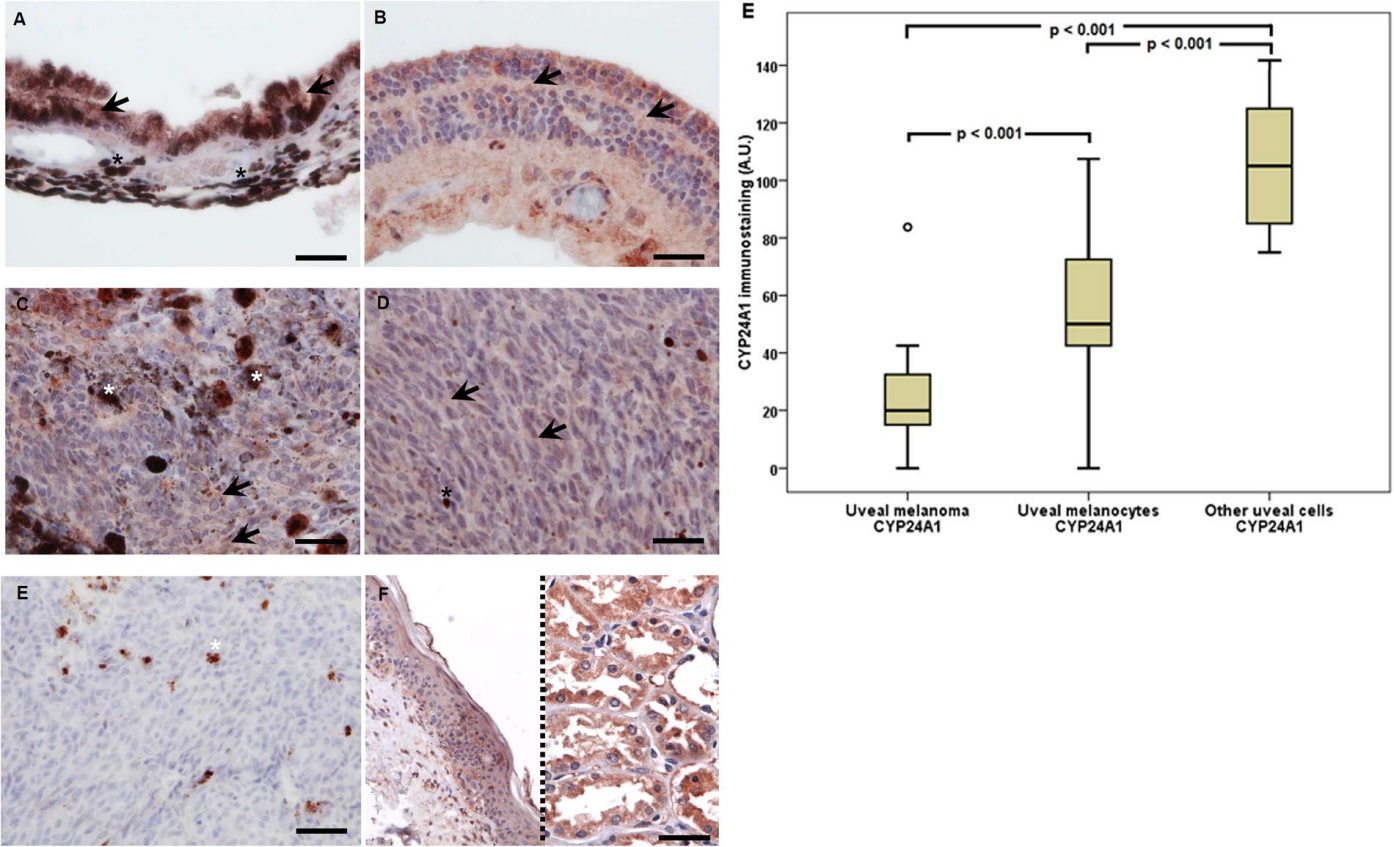
Representative CYP24A1 immunostaining in uveal pigmented cells (**A**), other cells (**B**), strongly pigmented (**C**) and slightly pigmented (**D**) melanomas and negative (**E**) and positive (**F**) controls. For CYP24A1 in addition to normal skin (left) also kidney (right) samples served as positive controls. Arrows indicate CYP24A1 immunostaining, asterisks indicate melanin, scale bar: 50 μm. Mean CYP24A1 immunolabelling in melanoma, normal melanocytes and other normal uveal cells (**F**) (statistically significant differences are denoted with the Wilcoxon signed-rank test).

CYP24A1, a mitochondrial monooxygenase belonging to the cytochrome P450 – superfamily, was found to be expressed in melanoma cells (90.32%), normal uveal melanocytes (67.74%) and other normal cells (54.84%) of the choroid coat (Fig. 2A–E). Mitochondrial monooxygenase is an enzyme, whose core function is the hydroxylation of an active form of 1.25-dihydroxyvitamin D_3_ into its inactive one. The lowest average values of this enzyme were found in the melanoma cells, larger ones – in normal melanocytes, whilst the largest – in other normal uveal cells.

The Wilcoxon signed-rank test showed that the immunostaining CYP24A1 distribution in uveal melanoma and uveal melanocytes, uveal melanoma and other uveal cells significantly differ from each other (p < 0.001) (Fig. 2F).

**Figure 3:**
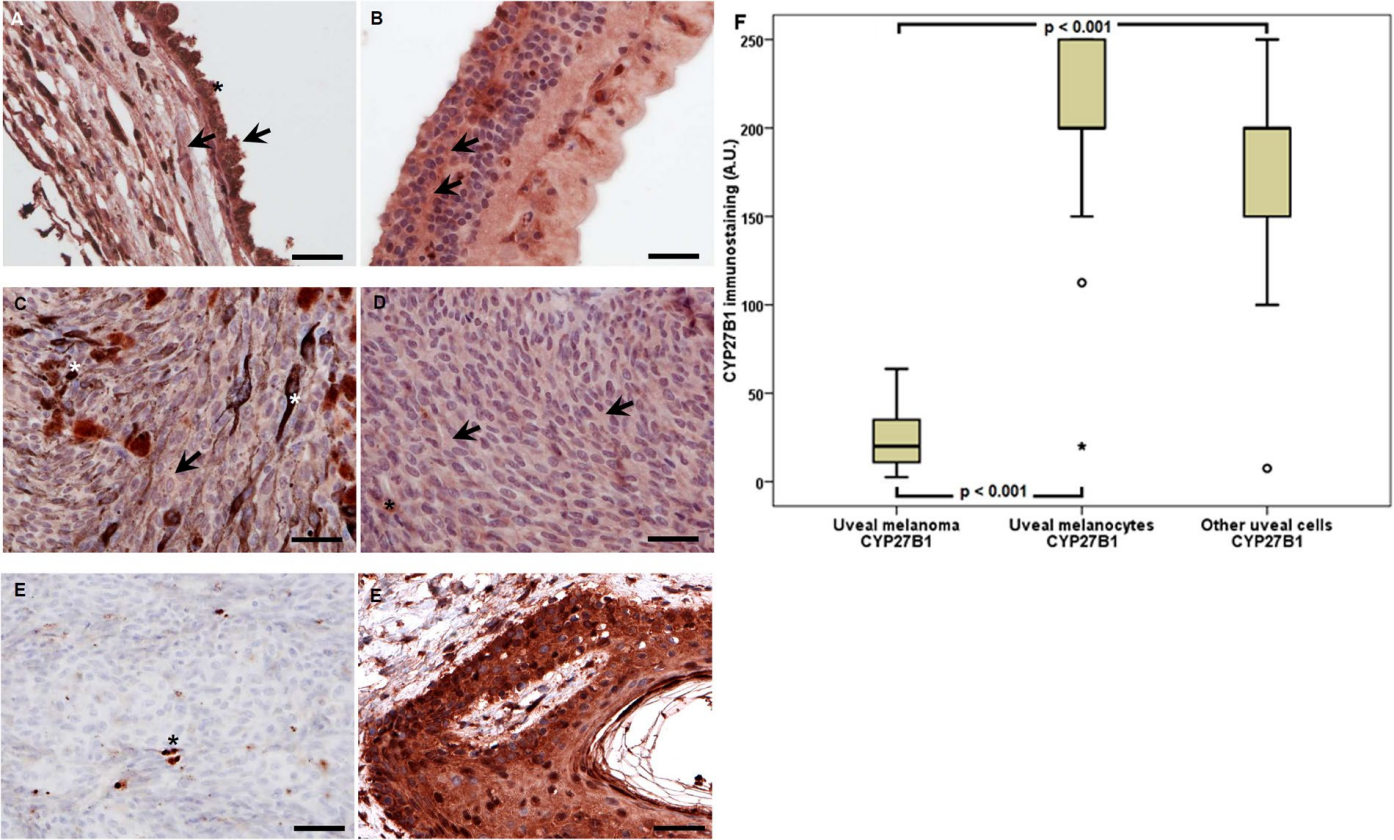
Representative CYP27B1 immunostaining in uveal pigmented cells (**A**), other cells (**B**), strongly pigmented (**C**) and slightly pigmented (**D**) melanomas and negative (**E**) and positive (**F**) controls. Arrows indicate CYP27B1 immunostaining, asterisks indicate melanin, scale bar: 50 μm. Mean CYP27B1 immunolabelling in melanoma, normal melanocytes and other normal uveal cells (**F**) (statistically significant differences are denoted with the Wilcoxon signed-rank test).

The presence of another enzyme belonging to cytochrome P450 superfamily, CYP27B1, and also known as 1-alpha-hydroxylase was found in 96.77% melanoma, 100% melanocytes, and in 70.97% other normal cells of the choroid coat (Fig. 3A–E). This enzyme catalyzes calcidiol (25(OH)D3) hydroxylation to calcitriol, i.e. 1,25(OH)_2_D3, the bioactive form of vitamin D.

In uveal melanoma, the values of CYP27B1 labelling were significantly lower than in normal melanocytes and other uveal cells (the Wilcoxon signed-rank test, p < 0.001) (Fig. 3F).

### RORα and RORγ in human uveal tract and uveal melanoma

**Figure 4:**
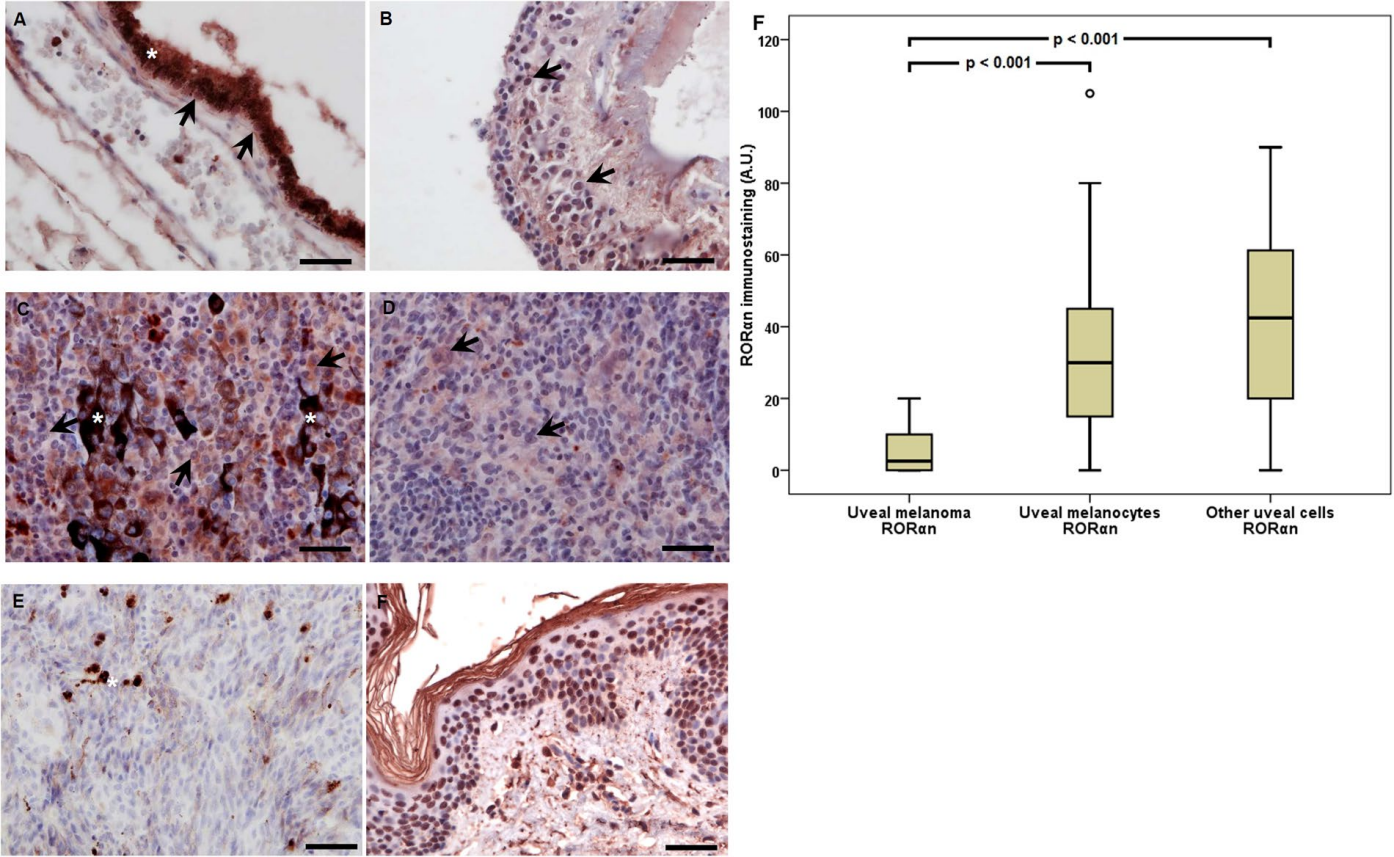
Representative RORα immunostaining in uveal pigmented cells (**A**), other cells (**B**), strongly pigmented (**C**) and slightly pigmented (**D**) melanomas and negative (**E**) and positive (**F**) controls. Arrows indicate RORα immunostaining, asterisks indicate melanin, scale bar: 50 μm. Mean RORα nuclear (RORαn) immunolabeling in melanoma, normal melanocytes, and other normal uveal cells (**F**) (statistically significant differences are denoted with the Wilcoxon signed-rank test).

Similarly, to cutaneous melanoma cells, RORα and RORγ were found to be expressed in both the nuclei (RORαn, RORγn), and the cytoplasm (RORαc, RORγc) of uveal melanoma and normal tissue. RORα and γ (NR1F1 and NR1F3, respectively) are member of the nuclear receptor superfamily^47^ that regulate gene transcription in many cell types. In our study, we observed expression of RORα in the nuclei (RORαn) of melanoma cells (93.55%), normal uveal melanocytes, (54.84%) and other normal uveal cells (64.52%) (Fig. 4A–E). The values of immunostaining of RORαn differed significantly between the tumor cells of the uveal melanoma, normal melanocytes and other uveal cells (the Wilcoxon signed-rank test, p < 0.001). Concerning differences between normal melanocytes and other normal uveal cells, there were no differences in immunostaining (the Wilcoxon signed-rank test, p = 0.557) (Fig. 4F). RORα immunostaining of the cytoplasmic component in the uveal melanoma cells was found in 93.55% of cells. Median was 2.50, and Q1 = 0.00 and Q3 = 7.50.

**Figure 5:**
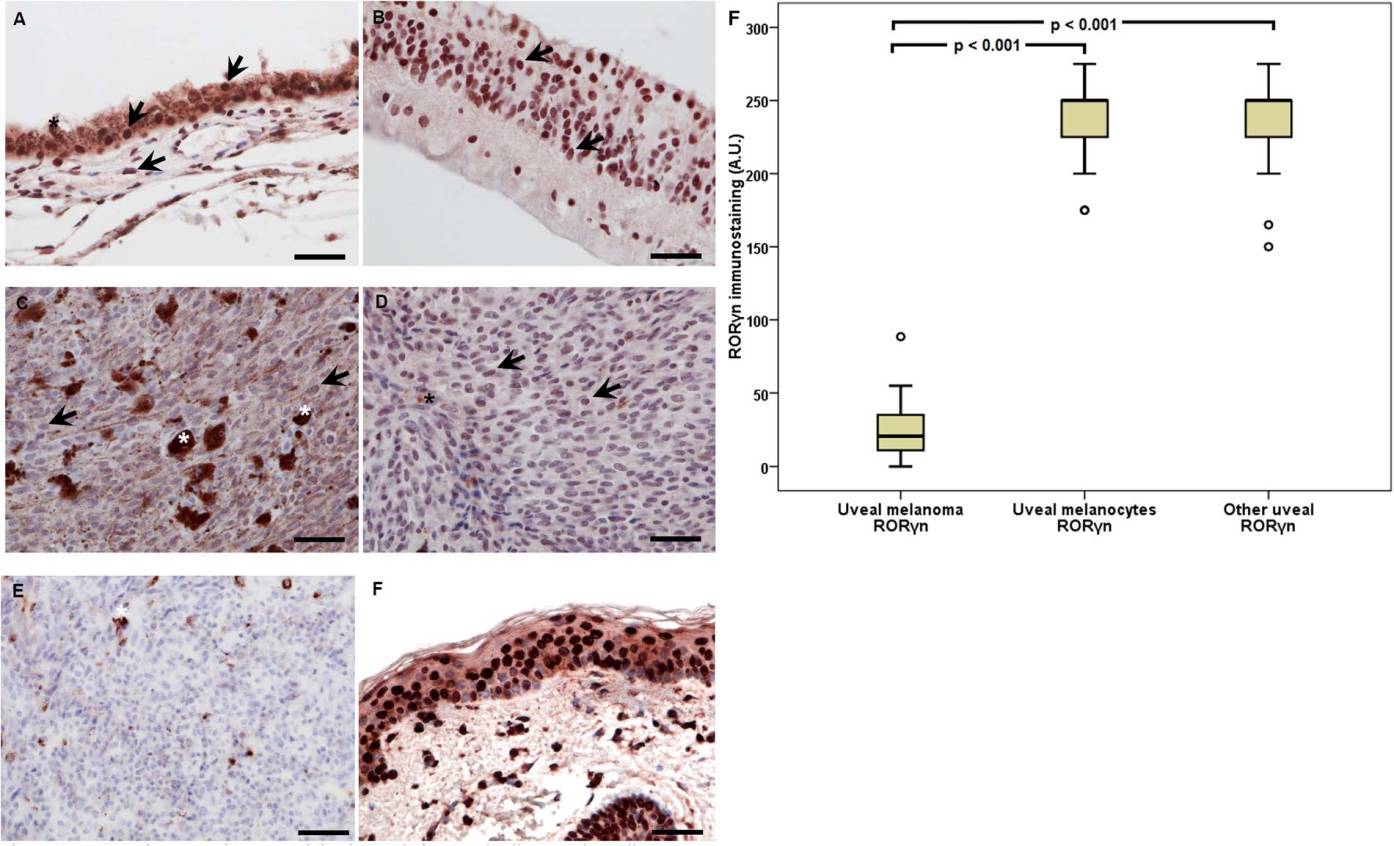
Representative RORγ immunostaining in uveal pigmented cells (**A**), other cells (**B**), strongly pigmented (**C**) and slightly pigmented (**D**) melanomas and negative (**E**) and positive (**F**) controls. Arrows indicate RORγ immunostaining, asterisks indicate melanin, scale bar: 50 μm. Mean RORγ nuclear (RORγn) immunolabelling in uveal melanoma, normal melanocytes and other normal uveal cells (**F**) (statistically significant differences are denoted with the Wilcoxon signed-rank test).

RORγ expression was observed in 93.55% of melanoma cells and in normal melanocytes (93.55%), as well as in other normal cells (77.40%) (Fig. 5A–E). Similar to RORα, the lowest level of nuclear RORγ was observed in the uveal melanoma cells. Nuclear immunostaining (median value) of this receptor differed between the tumor cells of the uveal melanoma and normal melanocytes and other normal uveal cells (the Wilcoxon signed-rank test, p < 0.001), with the lack of statistical significance between normal melanocytes and other normal uveal cells (the Wilcoxon signed-rank test, p = 0.718) (Fig. 5F). In uveal melanoma cells, also RORγc cytoplasmic immunostaining (93.55%) was observed. The median was 10.00, and Q1 = 5.00 and Q3 = 17.50.

### The correlation of VDR immunostaining and melanin in uveal melanoma

**Figure 6:**
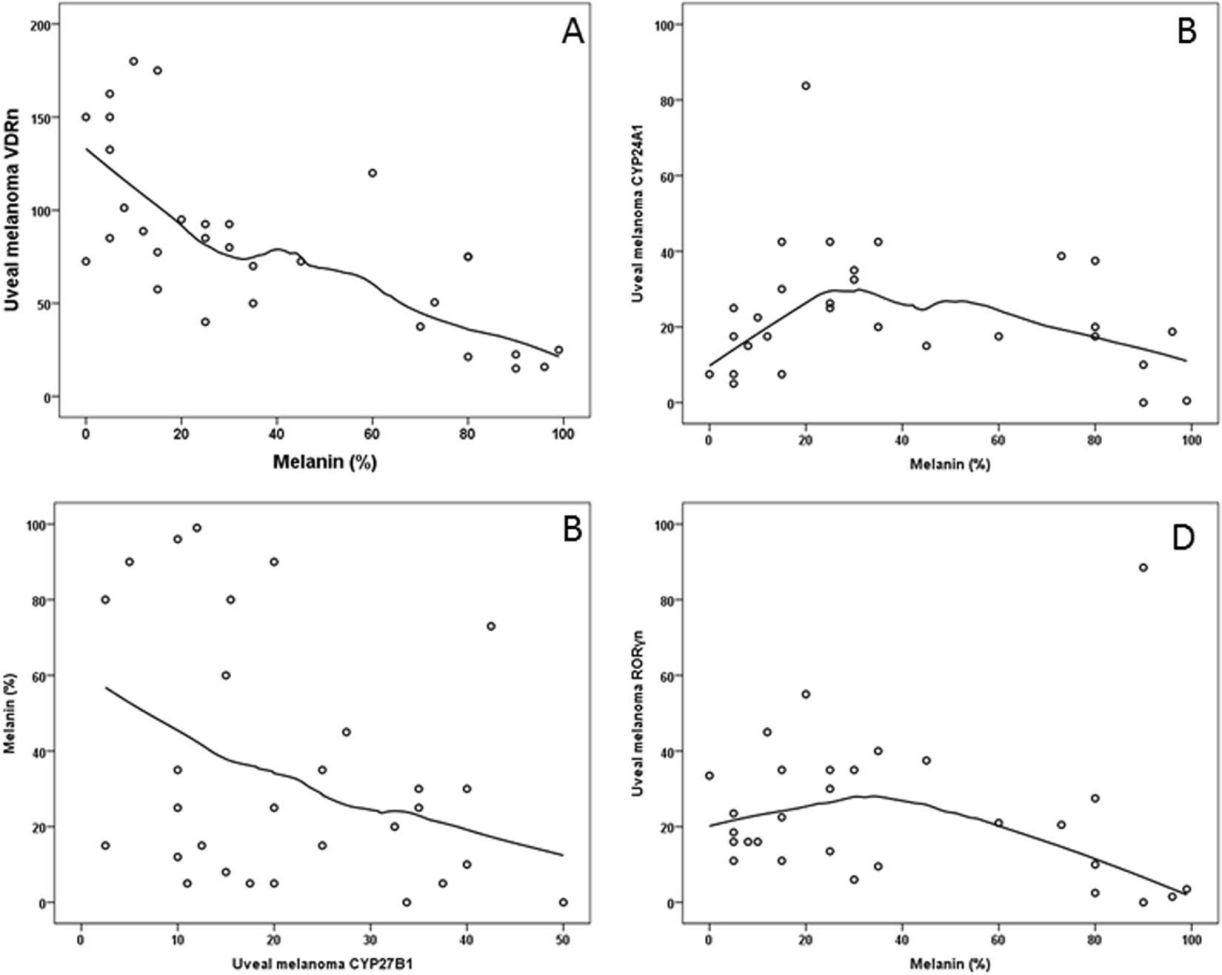
The correlation of: VDRn immunostaining and the level of melanin (**A**); CYP24A1 immunostaining and melanin (**B**); CYP27B1immunostaining and melanin (**C**); RORγn immunostaining and melanin (**D**).

The analysis of VDRn, VDRc and all VDR (VDRa = VDRn + VDRc) immunostaining and the degree of melanin pigmentation in uveal melanoma cells showed a negative correlation with the levels of VDRn and VDRa to melanin (Spearman’s rank correlation r = −0.704, p = 0.001 and r = −0.666; p = 0.004 respectively) (Fig. 6A). No such significant correlation was found for VDRc (r = −0.320, p = 0.079).

### Correlation between CYP24A1 and CYP27B1 immunostaining and melanin content in uveal melanoma

The correlation was statistically significant only for CYP24A1 expression in comparison to the melanin levels in uveal melanoma (p = 0.020). With low melanin levels, the level of CYP24A1 was the lowest (mean level = 14.6875, SD = 7.37243), reaching the highest values at medium melanin level (mean level = 33.5417, SD = 719.32698), and reversed with the highest levels of melanin (mean level = 17.8333, SD = 13.73806) (Fig. 6B).

No significant correlations between CYP27B1 labelling and melanin were found. The graph shows that CYP27B1 level has the trend to decrease when the melanin content increases, yet this is not statistically significant (rho = −0.30, p = 0.111) (Fig. 6C).

### Correlation between RORα and RORγ immunostaining and melanin content in uveal melanoma

The evaluation of RORα and melanin level in uveal melanoma did not reveal any significant correlations. Solely a tendency to reverse proportion (p = 0.576) was observed. For lower melanin values, the level of RORαn was increased.

The analysis of the correlation between RORγ immunostaining and melanin in the tumor cells showed statistical correlation, indicating that RORγn was higher in the tumors with a lower (<50%) melanin level (p = 0.030) (Fig. 6D).

### The relationship between VDRn, VDRc, VDRa and CYP24A1 and CYP27B1 in uveal melanoma

**Figure 7:**
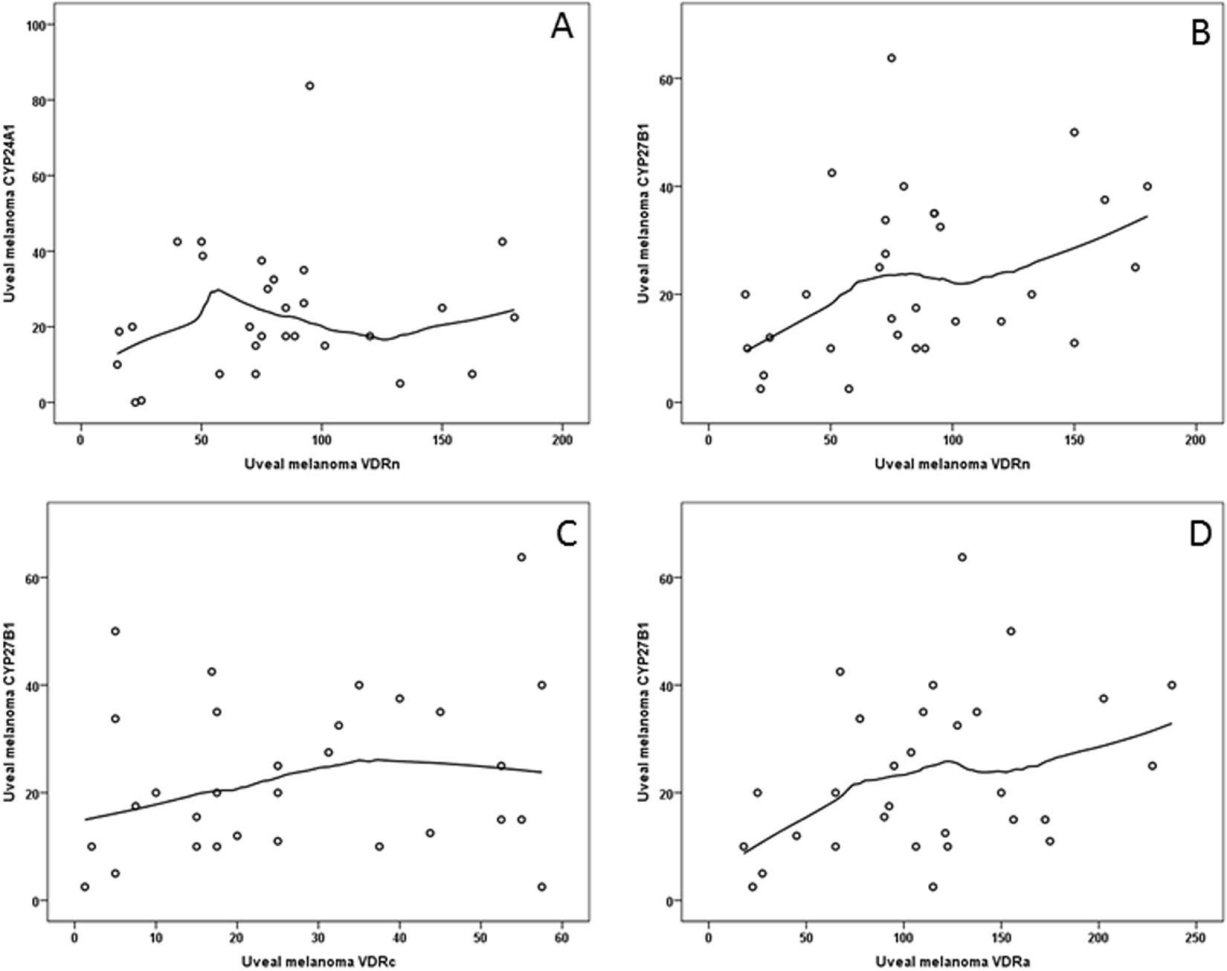
The correlation between VDRn immunostaining and CYP24A1 level (**A**). The correlation of the level of CYP27B1 to VDRn (**B**), VDRc (**C**) and VDRa (**D**).

No significant correlations between VDR and CYP24A1 in the cells of uveal melanoma were found. However, there was a tendency observed, indicating that together with an increase of VDRn to the level of about 40, the CYP24A1 value also increases (Fig. 7A).

The levels of VDRn and VDRa increased together with those of the CYP27B1 value (Spearman’s rank correlation, rho = 0.401, p = 0.028 and rho = 0.390, p = 0.033, respectively) in uveal melanoma cells. For VDRc a tendency of reverse correlation is observed, but without a statistical significance p = 0.254 (rho = 0.22) (VDRn, VDR and VDRa to CYP27B1) (Fig. 7B–D).

### Correlation between VDR immunolabeling and RORα and RORγ in uveal melanoma

The analysis of VDR and ROR immunostaining in the tumor cells revealed a statistically significant positive correlation between RORαn and VDRn in the melanoma cells (p = 0.030). No such correlation in the melanoma cells was found between RORαn and VDRc and VDRa (p = 0.476 and p = 0.118 respectively). In the melanoma cells, increased levels of RORγn correlated with increased levels of VDRn (p = 0.006) and VDRa (p = 0.022). No correlation was found between the levels of RORα or RORγ and CYP24 or CYP27.

### The relationship between VDR and clinical and pathomorphological features

Lack of statistically significant correlations were found between the levels of VDR (with one exception described above), CYP27B1, CYP24A1 and ROR, and histopathological diagnosis of uveal melanoma (spindle-cell melanoma, mixed and epithelioid cell-type), the degree of sclera infiltration, the degree of optic disc infiltration, the presence of tumor cell emboli in the scleral vessels, the involvement of the ciliary body, tumor size (the largest base diameter and height), tumor shape (mushroom-shaped, dome-shaped and diffuse), the clinical advancement stage (AJCC) and 2-year survival. Some tendencies could be observed, yet without statistical significance.

**Figure 8:**
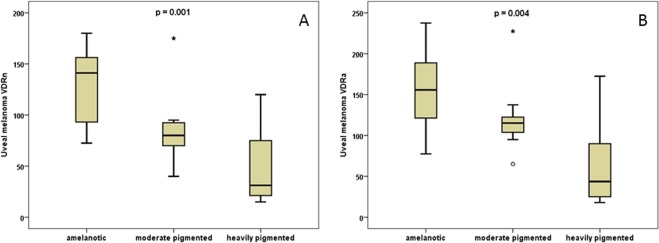
Comparison of VDRn (**A**) and VDRa (**B**) expression with the clinical assessment of tumor pigmentation (the Wilcoxon signed-rank test).

The only exception was the statistically significant correlation between the levels of VDRn and VDRa, and clinically assessed degree of tumor pigmentation (p = 0.001 and p = 0.004 respectively). In amelanotic tumors, the levels of VDRn were the highest, then VDRn decreased in medium-pigmented ones, whilst the lowest values were observed in the strongly-pigmented (dark brown) uveal melanomas (Fig. 8A). The same correlation was found for VDRa (Fig. 8B). No such correlation was found for VDRc (p = 0.072).

## Discussion

In this study, we provide evidence for the existence of vitamin D signaling in normal uveal cells (fibroblasts and melanocytes) and in tumors originating from this part of the eyeball in humans, i.e. uveal melanoma. Our study provides evidence showing the presence of VDR and CYB27B1 and CYP24A1, hydroxylases taking part in vitamin D metabolism, as well as RORα and RORγ, alternative receptors for vitamin D-hydroxyderivatives.

In all of the tissues examined the presence of nuclear receptors for vitamin D (VDRn), RORαn and RORγn, as well as CYB27B1 and CYP24A1, were confirmed. Moreover, cytoplasmic expression of VDR, RORα and RORγ (VDRc, RORαc and RORγc) was observed in the cells of uveal melanoma. The levels of VDR, CYP24A1, CYP27B1 and RORs were always the lowest in the cells of uveal melanoma and higher in normal uveal melanocytes and other normal uveal cells.

The remaining challenge is to establish molecular principles underlying the differences of protein expression between malignant and non-malignant samples. Since current study are focused on the paraffin embedded archival tissues, future studies on samples collected from the patients would clarify at which level these events are regulated, e.g., gene transcription, RNA processing, translation or posttranslational modifications.

With respect to other tumors, a similar decrease in the level of the above markers was observed in comparison to normal cells surrounding the tumor. In the case of breast cancer, Al.-Azhri *et al*. observed reduced VDR expression that was inversely correlated with the aggressive tumor characteristics, including large tumor size, hormonal receptor (HR) negativity, and triple-negative subtype (P < 0.05)^63,64^.

In ovarian cancers, normal ovarian epithelium showed the highest levels CYP27B1, opposed to a significant decrease of its expression in cancer tissue^31^. In urinary bladder cancer, the level of VDR and CYP27B1 in tumor tissues was significantly lower than in normal epithelium, which was observed by Jóźwicki *et al*.^29^. Bises and colleagues found a higher CYP27B1 expression in well-differentiated colon cancers compared with normal mucosa; however, in tumor areas showing a considerably poor differentiation, this expression was lost^56^. Also in skin melanoma, a significantly decrease in the expression of VDR, CYB27B1, CYP24A1, RORα and RORγ was observed in comparison to normal melanocytes or perilesional keratinocytes^28,62,65–67^.

In the current study a reverse correlation was found between the melanin level and VDR in uveal melanoma. The same correlation was observed when the degree of lesion pigmentation was analyzed in the clinical picture in comparison with the nuclear VDR. In predominantly amelanotic tumors, the levels of VDR were the highest, then VDRn decreased in medium-pigmented (dark brown) uveal melanomas. Similar observations were made in the studies cutaneous melanoma, where the level of VDR decreased together with an increase of melanin content and poorer prognosis^27,65^. It was also shown that melanogenesis induction in human SKMel-188 melanoma cells was accompanied by a marked decrease in VDR expression and responsiveness to active forms of vitamin D^26,68^. Several reports have indicated that in uveal melanoma, the risk of metastases increases with increased tumor pigmentation^7,69^. In the above context, the loss of the protective function of vitamin D could be secondary to the reduction of the VDR expression by an increased melanogenesis. Of note, melanogenesis can affect biological functions of melanoma cells^70–75^ and normal melanocytes^76,77^.

Vitamin D metabolism is connected to the CYPs expression, which catalyze the reaction in which the active form of vitamin D is formed or metabolized to biologically inactive forms. In our study we confirmed the presence of CYP27B1 and CYP24A1 in the melanoma cells and their connection with the VDR level. Together with an increase of the CYP27B1 value, the levels of VDRn and VDRa were increasing and, for VDRc, a tendency of a direct proportionality was observed. In the case of CYP24A1, together with an increase of VDRn in the melanoma to the level of about 40, the CYP24A1 value also increased. However, once this value was reached, there was a lack of statistical significance for any further correlation. The above results are similar to those reported for other tumors including cutaneous melanoma^66^.

Thus, the decreases in both CYP27B1 and VDR expression in uveal melanomas could reflect defects in the local vitamin D signaling pattern, which could facilitate melanoma progression and invasion. Similar findings were made by Brożyna *et al*. in a study on cutaneous melanomas^67^. Patients with low CYP27B1 and VDR expression had a shorter overall survival. Moreover, an absence of CYP27B1 or a reduction promoted the development of metastases and correlated with shorter disease-free survival^28,65,67^. A correlation was found between the level of CYP24A1 and melanin in uveal melanoma, yet no correlations were observed between the CYP27B1 labelling and melanin.

With respect to ROR expression in uveal melanomas, it was found that together with an increase of RORαn, the level VDRn increased significantly and, together with an increase of RORγn in melanoma, the levels of VDRn and VDRa (p = 0.022) also increased. No significant correlations between RORαn and VDRc or VDR and between RORγn and VDRc were found, yet some tendency of direct proportional correlation was observed in these cases.

Our analysis on the correlation between RORα and RORγ and the presence of melanin in uveal melanoma, showed only a tendency for reverse correlation for RORα. Thus, lower levels of melanin was associated with increased expression of RORαn. Likewise, RORγn was higher in the tumors with a lower level of melanin. These observation are in agreement with observations made in cutaneous melanomas the expression level of RORα and RORγ decreased in strongly pigmented melanomas^62^.

Vitamin D has the number of confirmed anti-tumor functions, acting through VDR, such as inhibition of cell proliferation, accelerated apoptosis, regulation of the cell cycle and differentiation, among others thanks to the presence of FOXO protein, cyclin-dependent kinase inhibitors like p21 or p27, insulin-like growth factor (IGF), transforming growth factor beta (TGFβ), and Wnt-β/catenin^24^. These also include anti-tumor effects in cutaneous melanomas^26,27^. Therefore, we propose that higher levels of VDR in uveal melanoma might be connected with a better prognosis in this disease. Taking into consideration that metastases of uveal melanoma, as opposed to cutaneous melanoma, can occur after many years, further observation of the reported patients during the follow-up period is required for definitive conclusions. The confirmation of the above pattern on a larger group of patients with simultaneous correlation with the clinical outcome are required to better define the VDR as a prognostic factor. This would open the way for creating new adjuvant therapies of uveal melanoma that would base on vitamin D derivatives, as it has been demonstrated for other tumors including cutaneous melanoma. Additional open questions are whether the levels of VDR, CYP27B1 and CYP24A1 and ROR in the tumor cells can also serve as reliable prognostic factors and in what way the level of VDR may affect the efficiency of vitamin D in inhibiting the tumor growth and aggressiveness.

In conclusion, we provide an evidence for the presence of the VDR, CYB27B1, CYP24A1, RORα and RORγ in uveal cells, which changes in uveal melanomas and suggest that these proteins are involved in clinical presentation and represent targets for novel therapeutic approaches.

## Materials and Methods

The study involved 31 adult patients in whom, on account of uveal melanoma, the eyeballs were enucleated during the period between 2013 and 2014 in the Department of Ophthalmology and Ocular Oncology, Medical College of Jagiellonian University in Kraków, Poland. For all patients, enucleation was the only way to treat the affected eye, with no previous local or systemic therapies. The study group comprised 19 women (61.30%) and 12 (38.70%) man, at the age ranging from 39 and 83 years (mean age: 62.9, SD = 10.8). The tissues of the enucleated eyeball were examined, with special attention paid to the medium layer, i.e. the choroid coat and to the primary tumor – melanoma originating from pigment cells (melanocytes) of this part of the eye. The disease affected the left eye more frequently (22 cases; 71.00%) than the right one (9 cases; 29.00%). The clinical TNM stage of the cancer was T3 in 17 cases (54.80%) cases and T4 in 14 cases (45.20%), whilst the AJCC stage was in 4 cases (12.90%) IIa, and in 13 cases – (41.90%) IIb, in 7 cases (22.60%) – IIIa, in 4 cases (12.90%) IIIb and in 3 cases (9,70%) IIIc^78^.

In the ultrasound picture, a mushroom-shaped tumor was dominating (19; 61.30%), followed by dome-shaped one (11; 35.50%), and only in 1 case (3.20%) a diffuse shape was observed. In all cases, histopathological assessment confirmed the diagnosis of uveal melanoma. In 25 cases (80.60%) it was melanoma of the choroid coat, in 4 cases (12.90%) of the choroid coat and ciliary body, and in 2 cases (6.50%) of the choroid coat, ciliary body and iris. On the basis of the modified Callender’s classification of uveal melanoma, the following histopathological subtypes of melanoma were diagnosed: spindle-cell – in 11 cases (35.50%), mixed – in 13 cases (41.90%) and epithelioid cell-type in 7 cases (22.60%). The eyeball infiltration and extra-bulbar infiltration were observed in 6 cases (19.40%) and in 5 cases (16.10%) cases respectively. Necrosis was found in 3 (9.75%) tumors. Optic disc involvement was observed in 11 cases (35.50%), and the involvement of ciliary body in 6 (19.40%) tumors.

The degree of tumor pigmentation (light, i.e. predominantly-amelanotic, medium-pigmented and strongly-pigmented, i.e. dark-brown) was assessed by 2 ophthalmologists working independently, during the enucleation procedure (after the resection, in order to collect the study specimen) and/or during an ophthalmoscopic assessment or on the basis of the evaluation of the photograph of the patient eye fundus. Additionally, for the purpose of further analysis, the percentage content of melanin in all the histopathological specimens was made, distinguishing 3 groups depending on the melanin level: 0–10 (predominantly-amelanotic), 11–50 (medium-pigmented) and 51–100 (with strong pigmentation). In all the cases, the clinical assessment and histopathological assessment overlapped. In the analyzed tumors, 8 predominantly-amelanotic types (25.80%), 13 medium-pigmented types (41.90%) and 10 types with strong pigmentation (32.30%) were found.

The follow-up period was 2 years. The analysis of the overall survival (OS) in this period found that 8 patients (25.80%) died during that period, out of whom 6 patients (19.40%) died of the dissemination of uveal melanoma, 1 person (3.20%) died of a different cancer and another 1 person (3.20%) died of a sudden cardiac arrest. In the evaluation of the disease free survival (DFS) in that period, no case of local recurrence in the orbit was found, but in 8 patients (25.80%), cancer dissemination was found on additional assessments. In all these cases, some numerous isolated metastases in the liver were found and, additionally, in 1 case (3.20%) melanoma foci were observed in the lungs.

In all the specimens, the tissue of uveal melanoma, normal melanocytes and connected tissue cells of the choroid coat were analyzed with regards to the presence of nuclear and cytoplasmic vitamin D receptors for and enzymes taking part in its metabolism, such as CYP27B1 and CYP24A1, in the manner described below. In these cells also the presence of RORα and RORγ was assessed.

After the presence of all the above markers was found, their qualitative and quantitative analyses were made and then, with the use of statistical analysis tools, the correlations between these markers and also between these markers and the clinical features (including pathomorphological ones) of uveal melanoma were evaluated.

Authors declare that this investigation was carried out following the rules of the Declaration of Helsinki of 1975 (revised in 2008) and this study was approved by the Ethics Committee of the Jagiellonian University, Krakow, Poland (No 1072.6120.34.2017, 20 June 2017). The Bioethical Committee of the Jagiellonian University (Poland), which gave their permission for conducting this study, waived the requirement to obtain Patients’ informed consent for this research.

### VDR, CYP27B1, CYP24A1, RORα and RORγ immunohistochemistry

VDR, CYP27B1, CYP24A1, RORα and RORγ expression was detected using immunohistochemistry as previously described^28,29,31,61,65,66,79^ with slight modifications. Briefly, formalin-fixed, paraffin-embedded 4µm-sections, after antigen retrieval in high pH buffer using PT-Link device (Dako, Glostrup, Denmark), were stained overnight using appropriate antibodies (Table 1). Imm-PACT NovaRED (Vector Laboratories Inc., Burlingame, CA, USA) was used as a substrate for peroxidase for visualization. Then, sections were counterstained with hematoxylin (except VDR immunostaining), and mounted under the non-aqueous medium (Consul Mount; Thermo Fisher Scientific Inc. Waltham, MA, USA). For negative controls primary antibodies were replaced with antibody diluent. Samples of normal skin served as positive control. In addition, for CYP24A1 kidney samples were used as additional positive controls.

**Table 1:** Description of the antibodies used and their manufacturers, and of immunocytochemistry protocols and detection methods.

Antibody	Vendor	Permeabilization/blocking before primary antibodies application	Primary antibody (dilution; incubation time)	Secondary antibody (dilution; incubation time; vendor)
Anti-VDR, rat monoclonal, clone 9A7	Abcam (Abcam Inc., Cambridge, MA, USA)	NA	1:50, O/N, 4 °C	anti-rat, peroxidase-conjugated goat antibody (1:250, 30 min, Jackson ImmunoResearch Laboratories, Inc. West Grove, PA, USA);
Anti-CYP27B1, rabbit polyclonal, clone H-90	Santa Cruz Biotechnology, Inc. (Santa Cruz, CA, USA)	0.2% Triton-X100, 15 min, RT	1:75, O/N, 4 °C	EnVision System-HRP (RTU, 30 min, antibody with peroxidase-conjugated polymer backbone,Dako, Carpinteria, CA, USA)
Anti-CYP24A1, mouse monoclonal,	Abcam (Abcam Inc., Cambridge, MA, USA)	0.2% Triton-X100 in 1% BSA, 25 min, RT	1:30, O/N, 4 °C	EnVision System-HRP (RTU, 30 min, antibody with peroxidase-conjugated polymer backbone, Dako, Carpinteria, CA, USA)
Anti-RORα, goat polyclonal, clone C-16	Santa Cruz Biotechnology, Inc. (Santa Cruz, CA, USA)	2% BSA, 20 min, RT	1:500, O/N, 4 °C	EnVision System-HRP (RTU, 30 min, antibody with peroxidase-conjugated polymer backbone,Dako, Carpinteria, CA, USA)
Anti-RORγ, rabbit	customer-made at NIEHS by Dr Jetten*	0.1% Triton-X100 in 3.5% BSA, 60 min, RT	1:50, O/N, 4 °C	EnVision System-HRP (RTU, 30 min, antibody with peroxidase-conjugated polymer backbone,Dako, Carpinteria, CA, USA)

RT – room temperature, O/N, overnight; NA – not applied; BSA- bovine serum albumin; RTU – ready-to-use; *for detailed description see^47,62^. The melanin content was assessed in routine pathomorphological slides (stained with hematoxylin and eosin) as previously described^62,79^.

Evaluation of immunostained sections has been performed by two independent observers in a blind manner, without knowing the detailed histopathological diagnosis and the clinical data. VDR, CYP27B1, CYP24A1, RORα and RORγ immunostaining of melanomas was scored semiquantitatively as previously described and both staining intensity, using a four-point scale, and percentage of cells was evaluated and calculated as follows: SQ = mean(IR × SI)/100, where IR is the percentage of immunoreactive cells and SI is the staining intensity (assessed using the scale from 0 to 3 arbitrary units (A.U.). The staining intensity in positive controls served as reference for staining intensity evaluation in samples of uveal melanoma samples. Since the immunostaining of CYP24A1 in normal skin samples were heterogenous between different samples, CYP24A1 were assessed in relation to its expression in the kidney^28,29,31,61,65,66,79^.

### Statistical analysis

The distribution of qualitative variables was described with the absolute numbers in specific categories (n) and their percentage distribution in that variable (%). Average values of variables with normal distribution were described with the mean value and standard deviation (SD), whilst in the case of variables whose distributions were significantly different from the normal ones – with the median and quartiles: the first and the third ones. The compliance of the distribution of a given variable with the normal distribution was assessed with the Shapiro-Wilk test, and with skewness and also it was visually evaluated by means of histogram analysis, boxplot and Q-Q plot (quantile-quantile).

Distributions of the studied variables were presented using boxplots, where bolded line at the interior of the box represents median, lower and upper end of the box (hinges) represent the 1^st^ and the 3^rd^ quartile, respectively. Whiskers represent observations ranging up to 1.5 of interquartile range from the box, circles represent observations ranging from 1.5 up to 3 interquartile ranges above or below from the hinges, and asterisks represent observations lying above or below 3 interquartile ranges from the hinges.

Relationships between particular variables were presented as scatterplots with added locally smoothed lowest curve, estimated using Epanechnikov algorithm.

The comparison of mean values of the variables with normal distribution in two nonrelated groups was made with the t-Student test for independent groups, whilst in more than two unrelated groups – with the ANOVA analysis of variance. The comparison of mean values of variables with distribution different significantly from normal ones in two unrelated groups was made with the Mann-Whitney test, whilst in more than two unrelated groups with the Kruskal-Wallis test. The comparison of mean values of the variables with the distributions different significantly from normal ones in two unrelated groups was made with the Wilcoxon test for related variables measured on interval or ordinal scale.

The strength of the correlation between the variables measured in interval scale was evaluated with the Pearson correlation coefficient, when between the variables there was a straight-line relationship, whilst, with Spearman’s Rho coefficient when the relationship was monotonous, but not a straight-line one, and in all other cases – with the eta correlation coefficient. A graphic presentation of the correlations between the analyzed variables was made with a scatterplot with the plugged loess curve, illustrating mean values of the variable presented on the Y axis and corresponding to the values of the variable presented in the X axis. The effects with the value p < 0.05 were regarded as statistically significant. The calculations were made with the IBM SPSS Statistics 24 for Windows software.
